# Local production of alcohol-based hand rub to optimize hand hygiene facility in healthcare settings during COVID-19

**DOI:** 10.1017/ash.2022.147

**Published:** 2022-05-16

**Authors:** Bobson Fofanah, Sia Tengbe, Ibrahim Kamara, Musa M. Korjie

## Abstract

**Background:** Hand hygiene (HH) remains arguably the most effective way to prevent healthcare-associated infections (HAIs) and ultimately improve the prospect of patient safety. Studies have shown that as many as 50%–70% of infections are transmitted through hands due to poor HH practices. HH with use of alcohol-based hand rub (ABHR) is preferred over handwashing with soap and water because of its wide microbial efficacy, time efficiency, and improved skin tolerance. It is also well known that ABHR can be used as an effective prevention measure during disease outbreaks. Before and during the COVID-19 pandemic, health facilities in Sierra Leone have been challenged with HH infrastructural problems such as lack of sinks with constant running water. Before Sierra Leone recorded its first case of COVID-19 in March 2020, the consumption of ABHR in the health facilities was estimated to be 24,000 L per year, which doubled during the COVID-19 pandemic. The demand for commercially available ABHR increased, leading to acute shortages. The estimated cost of the locally produced ABHR ~$2–3 per 500 mL, although it may cost up to $10 for 500 mL when buying imported ABHR products from the local market. **Methods:** All ingredients were procured locally, and ABHR production was based on WHO formula 1. The production was set for 12 months to cover the estimated annual consumption of ABHR, with periodic monitoring to ensure effective distribution and availability at the point of care. Analysis of assessment results in 12 hospitals from the pre-COVID-19 era (2019) to the COVID-19 era (2021) was performed based on the WHO IPC Assessment Framework (IPCAF) indicator. **Results:** With an average monthly production of 3,482 L, a total of 41,780 L ABHR was produced and packaged in branded 500-mL containers for distribution to healthcare facilities. This quantity exceeded the estimated demand for ABHR during the COVID-19 pandemic. The data show a considerable increase (from 25% to 44%) in the number of available and functioning HH stations with mainly locally produced ABHR. Results from the monitoring of 575 peripheral health units (PHUs) in 2021 also showed that >67% of PHUs had HH facilities in all clinical areas and that the locally produced ABHR was used in 79% of these HH stations. **Conclusions:** Locally produced ABHR has shown to be a cost-effective and evidence-based intervention to optimize HH at the point of care. Therefore, localities are encouraged to undertake this realistic and sustainable approach to address issues of acute shortage of ABHR, especially during a global pandemic.

**Funding:** None

**Disclosures:** None

